# Opportunistic screening for long-term muscle wasting in critically ill patients: insights from an acute pancreatitis cohort

**DOI:** 10.1186/s40001-024-01884-7

**Published:** 2024-05-22

**Authors:** Johannes Kolck, Clarissa Hosse, Alexandra Leimbach, Nick L. Beetz, Timo A. Auer, Federico Collettini, Uli Fehrenbach, Christian Pille, Dominik Geisel

**Affiliations:** 1 Department of Radiology, Charité - Universitätsmedizin Berlin, Corporate member of Freie Universität Berlin and Humboldt-Universität zu Berlin, Augustenburger Platz 1, 13353 Berlin, Germany; 2 Department of Anesthesiology and Intensive Care Medicine | CCM | CVK, Charité – Universitätsmedizin Berlin, Berlin, Germany; 3 Berlin Institute of Health at Charité - Universitätsmedizin Berlin, Berlin, Germany

**Keywords:** Critical care, Acute pancreatitis, Muscle wasting, Artificial intelligence, Computed tomography

## Abstract

**Objectives:**

To assess the feasibility of long-term muscle monitoring, we implemented an AI-guided segmentation approach on clinically indicated Computed Tomography (CT) examinations conducted throughout the hospitalization period of patients admitted to the intensive care unit (ICU) with acute pancreatitis (AP). In addition, we aimed to investigate the potential of muscle monitoring for early detection of patients at nutritional risk and those experiencing adverse outcomes. This cohort served as a model for potential integration into clinical practice.

**Materials:**

Retrospective cohort study including 100 patients suffering from AP that underwent a minimum of three CT scans during hospitalization, totaling 749 assessments. Sequential segmentation of psoas muscle area (PMA) was performed and was relative muscle loss per day for the entire monitoring period, as well as for the interval between each consecutive scan was calculated. Subgroup and outcome analyses were performed including ANOVA. Discriminatory power of muscle decay rates was evaluated using ROC analysis.

**Results:**

Monitoring PMA decay revealed significant long-term losses of 48.20% throughout the hospitalization period, with an average daily decline of 0.98%. Loss rates diverged significantly between survival groups, with 1.34% PMA decay per day among non-survivors vs. 0.74% in survivors. Overweight patients exhibited significantly higher total PMA losses (52.53 vs. 42.91%; *p* = 0.02) and average PMA loss per day (of 1.13 vs. 0.80%; *p* = 0.039). The first and the maximum decay rate, in average available after 6.16 and 17.03 days after ICU admission, showed convincing discriminatory power for survival in ROC analysis (AUC 0.607 and 0.718). Both thresholds for maximum loss (at 3.23% decay per day) and for the initial loss rate (at 1.98% per day) proved to be significant predictors of mortality.

**Conclusions:**

The innovative AI-based PMA segmentation method proved robust and effortless, enabling the first comprehensive assessment of muscle wasting in a large cohort of intensive care pancreatitis patients. Findings revealed significant muscle wasting (48.20% on average), particularly notable in overweight individuals. Higher rates of initial and maximum muscle loss, detectable early, correlated strongly with survival. Integrating this tool into routine clinical practice will enable continuous muscle status tracking and early identification of those at risk for unfavorable outcomes.

## Introduction

Among the many gastrointestinal conditions that lead to hospitalization, acute pancreatitis is one of the most common. The severity of acute pancreatitis (AP) encompasses a wide spectrum, ranging from clinically self-limiting cases to rapidly fatal courses [1, 2]. In severe cases, patients often necessitate intensive care unit (ICU) admission for comprehensive management*,* involving vigilant monitoring of consciousness, respiratory and cardiovascular function, urinary output, along with appropriate fluid replacement and pain control [3]. While multidisciplinary approaches often remain essential, treatment approaches in severe pancreatitis have shifted to emphasize aggressive intensive care over early surgical intervention [4].

In this setting, providing nutritional support is one of the cornerstones of management. In general, patients with severe AP are recognized as being at nutritional risk and malnourished individuals afflicted with AP are more likely to encounter worse outcomes. During hospitalization, the assessment of patients’ nutritional status can be difficult, as traditional anthropometric and biochemical markers may be challenging to obtain, often resulting in insufficient nutritional support. In contrast, changes in tissue mass are considered a more reliable indicator [5, 6].

Tracking muscle deterioration in critically ill patients has been focus of previous studies, as severe loss of muscle mass and function resembles a common complication of critically illness [7, 8]. Common techniques to estimate and track muscle mass include bioelectrical impedance analysis (BIA) and ultrasound (US), while tissue segmentation based on cross-sectional imaging, offering potentially more objective and precise quantification, has only been applied in very few studies [7]. In pancreatitis, imaging represents one of the three main pillars of diagnosis and is frequently used to determine the cause and assess the severity of the condition. In addition, it is recommended to repeat imaging studies, if the clinical picture worsens, to detect any underlying complications [8]. The increased utilization of cross-sectional imaging makes muscle monitoring with segmentation tools viable in this population.

The primary objective of this study was to evaluate the viability of long-term muscle monitoring based on clinically indicated examinations of patients with severe pancreatitis, using this cohort as a model for potential implementation in clinical practice. In addition, we aimed to assess the potential of muscle monitoring for early detection of patients at risk for adverse outcomes.

## Materials and methods

### Study design and patient population

In this retrospective cohort study, we retrospectively analyzed body composition metrics in critically ill patients admitted due to severe pancreatitis. The study was approved by the Institutional Review Board (Internal registration number: EA4/152/20) and conducted according to the principles of the Declaration of Helsinki. The study aims to evaluate the general applicability of image segmentation as a tool for muscle monitoring in critically ill acute pancreatitis patients, and to assess the value of the muscle loss rates obtained for outcome prediction. It is recognized that for such feasibility studies, sample sizes of 30 individuals per group (here survival vs. non-survival) are considered appropriate [3]. Therefore, we targeted a study size of > 60 patients. We retrospectively enrolled patients (> 18 years) who were admitted to the intensive care unit for at least 10 days and underwent at least three serial CT scans including the abdomen between 2012 and 2022. Patients were excluded, if they did not receive a CT scan prior or at the latest 6 days after ICU. The ethics committee waived the need to obtain patient consent. The study was conducted in accordance with the STROBE guidelines [9].

### Segmentation of tissue compartments

Quantification of patient’s tissue compartments was performed using applying an AI-based automated image segmentation tool, which is integrated into the hospitals Picture Archiving and Communication System (PACS) software (Visage version 7.1., Visage Imaging GmbH, Berlin, Germany). This tool was validated in previous studies, while tissue segmentation of CT scans at the level of the third lumbar vertebra (L3) is considered a gold standard for muscle assessment [10–12]. After automatically identifying the L3 level, the system conducted automated segmentation to distinguish tissues into subcutaneous fat (SAT), skeletal muscle area (SMA), visceral fat (VAT), and psoas muscle area (PMA). The software then computed the areas in square centimeters (cm^2^) for each of these four components. The Total Abdominal Muscle Area (TAMA) was determined by adding the SMA and PMA. An experienced radiologist reviewed each automated segmentation and made manual corrections if deemed necessary.

### Statistics

Descriptive statistics were computed as mean and 95% confidence interval (CI) to describe the collective (demographic data, hospitalisation, treatment, etc.). To evaluate muscle decay rates of the patients, absolute muscle loss per day was computed for the entire hospital stay (from the first to the last CT) as the difference between recorded values for psoas muscle area (in cm^2^) from the first and last CT examination divided by the intervening time interval (in days). Relative muscle decline per day was calculated by dividing the absolute psoas muscle loss per day by the baseline PMA of the first available scan (in cm^2^). Similarly, the maximum muscle loss between two consecutive CT scans and the loss between the first two scans were calculated in the same manner. The percentage of relative muscle loss between the initial and final scans, irrespective of the intervening days, was provided as an additional metric.

For subgroup analysis, independent *T* test and analysis of variance (ANOVA) were utilized to compare patient groups based on survival (overall and 100-day survival) and the underlying cause of pancreatitis.

Receiver operating characteristic (ROC) analysis was conducted to assess the sensitivity and specificity of relative psoas muscle loss (first, maximum, and overall) in predicting survival outcomes. The Youden index was computed to determine a potential cutoff for the relevant muscle decay rates. Kaplan–Meier curves were generated to assess survival probabilities or outcomes based on specific survival discriminators. Statistical analyses were performed using Stata/MP version 16 (StataCorp, College Station, Texas, USA) and SPSS Statistics 27 (IBM, Armonk, NY, USA). We classified overweight and obesity using internationally recognised BMI thresholds: BMI > 25 kg/m^2^ for overweight and BMI > 30 kg/m^2^ for obesity. Sarcopenia was determined using gender-specific cutoffs; SMA < 34.3 cm^2^ for women, and < 45.4 cm^2^ for men, based on established literature [13]. Pancreatitis types were categorized into five groups: post-ERCP, ethyl-toxic, biliary, other (including mainly post-surgical), and unknown. For patients transferred to our hospital during their illness, we extracted the timepoints of initial admission and ICU admission from the transfer letter. In the statistical analysis, these patients were treated in the same manner, as in-house patients. All *p* values less than 0.05 were considered statistically significant.

## Results

### Demographic data and preconditions

**Fig. 1:**
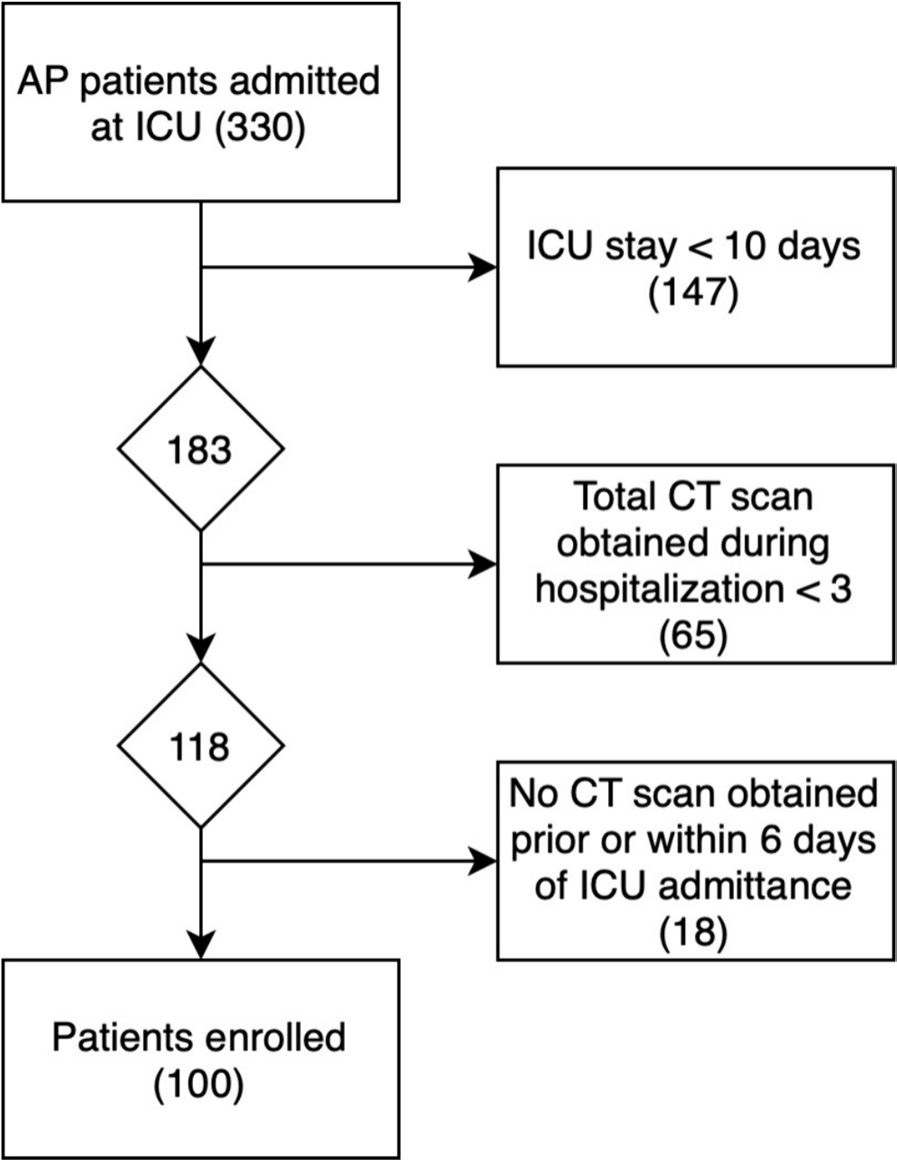
Flow chart of patient collection

Out of the 330 patients admitted to the ICU for AP, a total of 230 patients were excluded from the study. A total of 100 critically ill patients (75 men and 25 women), that underwent.a total of 749 CT scans, met the defined criteria and were retrospectively enrolled (Fig. 1).Mean age of the study population was 60.19 ranging from 19 to 94 years at the timepoint of hospital admission. Mean BMI was 26.27 kg/m^2^, ranging from 16.67 to 52.59 kg/m^2^. Forty-two patients (42%) had sarcopenia at admittance, fifty-five (55%) were obese, defined as BMI > 25 kg/m^2^, and nineteen (19%) were obese, defined as BMI > 30 kg/m^2^ (Table 1).The majority of patients (86%) presented with chronic pre-existing conditions prior to the acute pancreatitis. Among these, arterial hypertension was the most prevalent, affecting 49 out of 100 patients, followed by other cardiovascular conditions in 34 out of 100 patients, metabolic conditions in 21 out of 100 patients, pulmonary conditions in 14 out of 100 patients and malignant conditions in 9 out of 100 patients. Only five patients were pre-diagnosed with chronic pancreatitis. Grouped according to the etiology of pancreatitis, our study cohort comprised various categories: 22 patients with post-ERCP pancreatitis, 23 patients with ethyl-toxic pancreatitis, 23 with biliary pancreatitis, 10 with unknown reason for pancreatitis and 19 patients with other causes of pancreatitis. Among the latter, 10 patients developed pancreatitis post-surgically, 4 due to toxic causes, 2 post-traumatic cases, one associated with lipid metabolism disorder, one autoimmune, and one after the rupture of a pancreatic pseudocyst. These were summarized into a group named post-surgical and other. Notably, the exact cause of pancreatitis remained unclear in 13 patients despite extensive investigations. The overall survival (OS) rate was 59%, while 41 patients died during hospitalization.

**Table 1:** Descriptive data of patient collective

Collective	
Total patients	100
Gender	
Male	75
Female	25
Age (years)	60.19 (19–94)
BMI (kg/m^2^)	26.27 (16.67–52.59)
Obesity (BMI > 25 kg/m^2^)	55%
Sarcopenia at admittance	42%
Obesity (BMI > 30 kg/m^2^)	19%
Pre-existing conditions	
Arterial hypertension	49
Other cardiovascular	34
Metabolic	21
Pulmonary	14
Malignant	9
Hospitalization	
Hospital Length of Stay (days):	116.63
ICU Length of Stay (days):	82.39
SOFA Score at ICU Admission:	8.44 ± 5.44

### Hospitalization, complications and interventional treatment

Mean hospital length of stay was 116.63 days, with 82.39 days at the ICU. Average SOFA (Sequential Organ Failure Assessment) at ICU admission was 8.44 ± 5.44. During hospitalization, 64 patients exhibited renal failure and had to undergo dialysis. Most patients (88%) required invasive mechanical ventilation at some point during hospitalization, more than half (66%) had to undergo tracheostomy and 7 patients required extracorporeal membrane oxygenation (ECMO). Regarding pancreatic complications, 12 individuals exhibited abdominal compartment, 23 patients presented with bowl ischemia and 20 with pancreatic fistulas. Just over half of the patients (51%) experienced bleeding during their stay, 25 of which were managed by angiography. Peripancreatic fluid collections were managed through a multidisciplinary approach. On average, 57 patients underwent 2.96 open necrosectomies/lavages, while 43 patients received an average of 3.27 transgastric necrosectomies and 3.46 transgastric drainages. Furthermore, 58 patients underwent an average of 2.74 endoscopic retrograde cholangiopancreatography (ERCP) procedures, and 76 patients were subject to an average of 2.98 CT drainages (Table 2).

**Table 2:** Complications and interventional therapies in patients with acute pancreatitis who require intensive medical care during their stay

Complications	
Dialysis	64%
Invasive mechanical ventilation	88%
Tracheostomy	66%
Extracorporeal membrane oxygenation (ECMO)	7%
Pancreatic complications	
Abdominal compartment syndrome	12%
Bowel ischemia	23%
Pancreatic fistulas	20%
Bleeding episodes	51%
Managed by angiography	25%
Interventions (Average per patient)	
Open necrosectomies	2.96
Transgastric necrosectomies	3.27
Transgastric drainages	3.46
Endoscopic retrograde cholangiopancreatography (ERCP)	2.74
CT Drainages	2.98

### Initial analysis of muscle distribution

Average SMA of the collective was 140.16 cm^2^, ranging from a minimum of 73.53–226.82 cm^2^ and average PMA was 15.96, ranging from 4.39 to 32.0 cm^2^, resulting in an average TAMA of 154.99 ranging from 40.41 to 246.56 cm^2^. The collective’s mean VAT and SAT were 168.44 and 226.76 cm^2^, respectively. Upon admission, there were no statistically significant disparities observed in muscle distribution between the groups of patients who ultimately survived and those who did not: PMA was 15.87 cm^2^ in the survival group and 16.10 cm^2^ in the non-survival group. Similarly, the average SMA was 142.01 cm^2^ in the survival group and 137.50 cm^2^ in the non-survival group, resulting in a mean TAMA of 157.88 cm^2^ and 153.60 cm^2^, respectively.

### Muscle loss during hospitalization

**Fig. 2:**
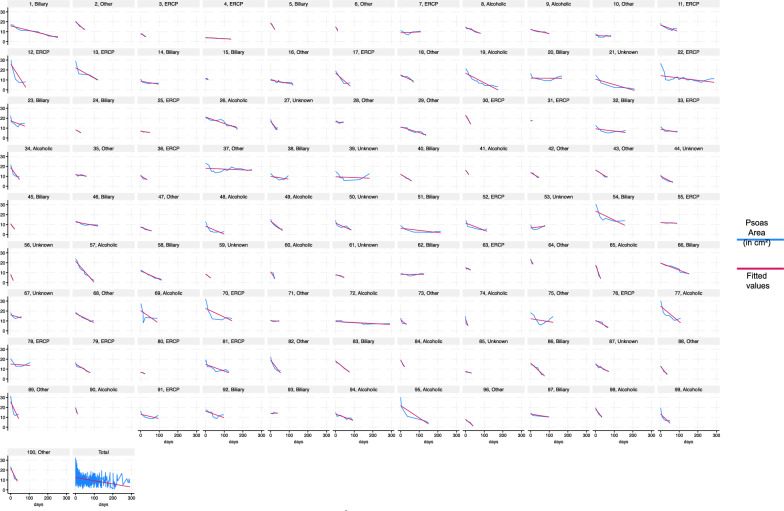
Comprehensive overview depicting the progression of muscle wasting in all one hundred ICU patients with acute pancreatitis. The psoas muscle area was segmented based on clinically indicated CT scans using an AI tool

Reviewing the automated segmentations uncovered instances of SMA swelling during hospitalization, which was attributed to fluid therapy and capillary leakage associated with sepsis. Notably, the assessment of PMA appeared less influenced by fluid accumulation. None of the obtained scans needed to be excluded from segmentation due to poor quality or technical obstacles. Utilizing segmentation data, distinct rates of relative muscle loss were computed concerning PMA and the duration between measurement intervals. The observed total muscle loss showed a non-linear trajectory, characterised by an overall negative trend with different rates of decline at different timepoints. A subgroup analysis of the patient collective was performed to gain a more comprehensive insight of muscle changes over time:

The mean long-term PMA loss for the entire cohort was 48.20% (CI 33.87–58.73%). Measurement of mean total/long-term PMA loss per day, calculated between the first and last CT scan, averaged 0.98% per day (CI 0.82–1.14%). Interestingly, in the majority of cases (59 out of 100), the peak of muscle wasting did not occur during the first measurement interval, in average 6.16 days after ICU admission, it occurred later, at an average of 17.03 days after admission to the ICU. The timeframe for experiencing the peak loss was notably wide-ranging: in 8 instances, it had already taken place before ICU admission, while in five cases, it manifested more than 50 days after admission to the ICU. The mean first loss was 2.33% per day (CI 1.73–2.93%), while the mean maximum decay accounted for 4.63% per day (CI 3.38–5.87%). Figure 2 provides an overview of the muscle status of each of the 100 patients.

#### Overall survival

In the cohort of 100 patients, decedents (41 patients) experienced a significant mean loss of 55.45% (CI 40.17–70.73%), while survivors (59 patients) displayed an average loss of 39.94% (CI 21.55–58.73%). Long-term PMA loss per day significantly differed between the survival groups (*p* < 0.01), with rates of 0.74% per day (CI 0.62–0.85%) in survivors and 1.34% per day (CI 1.00–1.67%) in non-survivors. Initial loss was notably higher in non-survivors (3.02% per day, CI 1.73–2.93%) than survivors (1.86% per day, CI 1.43–2.26%; *p* = 0.058). However, the mean maximum loss did not significantly differ between the survival groups (*p* = 0.22), recording a mean rate of 5.54% per day (CI 4.01–7.07%) in non-survivors and 3.51% per day (CI 2.53–4.49%) in the survival group.

#### 100-Day survival

Assessing survival within the initial 100 days revealed significant distinctions: the non-survivor group (29 patients) exhibited a substantial mean loss of 56.00% (CI 48.75–63.25%), contrasting with the mean loss of 45.02% (CI 40.14–49.91%) observed in survivors (71 patients). Notably, non-survivors experienced a higher mean daily loss of 1.61% (CI 1.17–2.05%) compared to survivors, who displayed a lower mean daily loss of 0.73% (CI 0.63–0.83%; *p* < 0.001). Specifically, the initial loss in non-survivors was notably higher at 3.54% per day (CI 1.73–5.34%) than in survivors, who had a mean loss of 1.84% (CI 1.43–2.26%; *p* = 0.022) per day. The differences in maximum loss rates between the groups accounted for a mean loss rate of 5.91% per day (CI 3.86–7.97%) in non-survivors, notably higher than the rate of 3.71% (CI 2.84–4.57%) per day observed in survivors.

#### Sarcopenia and overweight

Patients with sarcopenia (*n* = 42) naturally presented with lower SMA (117.85 vs. 156.31 cm^2^; *p* < 0.001) and PMA (12.56 vs. 18.42 cm^2^; *p* < 0.001), but also had significantly smaller SAT (193.23 vs. 251.04 cm^2^; *p* = 0.024) and VAT (137.64 vs. 190.73; *p* = 0.019*).* Sarcopenic patients experienced significantly lower total PMA loss of 40.23% vs. 53.97% in non-sarcopenic patients (*p* < 0.001), while the loss rate per day (0.98% vs. 1.02%) did not differ significantly (*p* = 0.59). Overweight patients (BMI > 25 kg/m^2^; *n* = 55) had significantly greater VAT (207.26 vs. 120.97 cm^2^; *p* < 0.001) and SAT (266.13 vs. 178.64 cm^2^; *p* < 0.001), but also greater SMA (151.12 vs. 126.76 cm^2^; *p* < 0.001). These patients were observed with significantly higher total PMA losses (52.53% vs. 42.91%; *p* = 0.02) and average PMA loss per day (of 1.13% vs. 0.80%; p = 0.039). Patients suffering from obesity (BMI > 30 kg/m^2^; *n* = 19) did not present significantly different rates of muscle decay, but had significantly higher SAT and VAT (*p* < 0.001; *p* = 0.11) values and exhibited significantly reduced survival rates (37% vs. 64%; *p* = 0.029).

#### Etiology of pancreatitis

The patient collective was subdivided into five groups depending on the underlying cause of the pancreatitis, namely, post-ERCP (group 1, *n* = 22), ethyl-toxic (group 2, *n* = 23), biliary (group 3, *n* = 23), post-surgical and other (group 4, *n* = 19) and unknown (group 5, *n* = 13). ANOVA analysis of these groups revealed significant differences survival (*p* = 0.006), gender and age distribution (*p* = 0.038 and *p* = 0.002), initial PMA (*p* = 0.23) and SMA (*p* = 0.046), as well as maximal PMA loss per day (*p* = 0.046) and relative overall/long-term loss per day (*p* = 0.037).

In detail, the survival rates in the biliary group was significantly decreased to only 30%, in contrast to the post-ERCP group (68%, *p* = 0.008) and the ethyl toxic group (83%, *p* < 0.001). However, there were no significant differences in survival compared to group 4 (58%, *p* = 0.61) and group 5 (54%, *p* = 0.153). Patients with post-ERCP pancreatitis had significantly less muscle mass (TAMA) on average compared to the ethyl-toxic (*p* = 0.023) and biliary group (*p* = 0.008). In addition, patients with biliary pancreatitis had more visceral adipose tissue (VAT) compared to patients with post-ERCP (*p* = 0.034) and ethyl-toxic pancreatitis (*p* = 0.051).

Analysis of muscle loss rates between groups showed that patients in the biliary pancreatitis group (1.22%; *p* = 0.028 and *p* = 0.039) and those with unknown cause of pancreatitis (1.38%, *p* = 0.015 and *p* = 0.021) had significantly higher average daily PMA losses than patients in the post-ERCP (0.70%) and ethyl-toxic (0.74%) groups. Conversely, patients with post-surgical and other pancreatitis (group 4; 1.04% PMA loss per day) showed no significant difference from the other groups. In terms of maximum muscle loss observed between two scans, the biliary group showed a notable difference, with an average PMA loss of 7.79% per day. This exceeded the maximum rates of group 1 (2.46%, *p* = 0.004), group 2 (3.35%, *p* = 0.015), group 4 (5.02%, *p* = 0.145) and group 5 (4.38%, *p* = 0.149). Compared to the other groups, the biliary pancreatitis group had the oldest population and the lowest proportion of women, with a mean age of 68 (CI 60.12–75.88) and only 2 female patients. There were no significant differences in complications and interventional treatment approaches between the groups. Relevant inter-group distinctions are compiled in Table 3, 4, 5*.*

**Table 3:** Comparison of muscle decay rates in survivors and non-survivors each for overall and 100-day survival

Category		n	Long-term Loss (%)	AVG Loss rate (%)	First Loss rate (%)	Max Loss rate (%)
OS	NS	41	55.45	1.34	3.02	5.54
	S	59	39.94	0.74	1.86	3.51
100-day Survival	NS	29	56	1.61	3.54	5.91
	S	71	45.02	0.73	1.84	3.71

*AVG* average

**Table 4:** Comparison of tissue distribution and muscle decay rates according to preexisting sarcopenia and overweight

Group	n	SMA (cm^2^)	SAT (cm^2^)	VAT (cm^2^)	Total Loss (%)	AVG Loss rate (%)
Sarcopenia	42	117.85	193.23	137.64	40.23	0.98
Non-Sarcopenic	58	156.31	251.04	190.73	53.97	1.02
Overweight	55	151.12	266.13	207.26	52.53	1.13
Normal Weight	45	126.76	178.64	120.97	42.91	0.8

*AVG* average

**Table 5:** Comparison of muscle loss rates according to the etiology of the severe pancreatitis

Group	Underlying cause	n	Survival rate (%)	Female (n)	Mean age	AVG Loss rate (%)	Max. Loss Rate (%)
1	Post-ERCP	22	68	10	65.86	0.70	2.46
2	Ethyl-Toxic	23	83	4	51.48	0.74	3.35
3	Biliary	23	30	2	68.00	1.22	7.79
4	Other	19	58	7	54.89	1.04	5.02
5	Unknown	13	54	3	64.77	1.38	4.38

### Outcome analysis

Comparing means between OS groups (59 survivors and 41 non-survivors) revealed significant variables from the spectrum of pre-existing conditions, disease complications and muscle loss rates. In particular, overweight showed a significant association with reduced survival (47.46% of overweight individuals among the survivors vs. 65.85% among the non-survivors, *p* = 0.017), while obesity showed an even stronger correlation with reduced survival (11.86% among the survivors vs. 29.27% among the non-survivors, *p* < 0.001). Similarly, cardiovascular disease was more common in non-survivors (28.81% vs. 41.46%, *p* = 0.022). Looking at complications, the non-survivors had significantly more intestinal ischemia (31.71% vs. 17.24%, *p* = 0.001) and dialysis requirement (75.61% vs. 56.90%, *p* < 0.001). There were no significant differences in hospital- and ICU-length of stay with 134.89 and 90.27 days in the survivor and 90.34 and 71.05 days in the non-survivor group. The average muscle loss per day diverged significantly between the survival groups, with 1.34% decay per day in the non-survivor and 0.74% in the survivor group (*p* < 0.001), whereas the first obtained loss with 1.86% vs. 3.02% did not differ significantly (*p* = 0.075).

Despite slight differences in numbers, the overall pattern remained similar when comparing the 71 survivors and 29 non-survivors in terms of 100-day survival. The survivor group comprised a significantly lower number of overweight (47.89% vs. 72.41%) and obese (12.68% vs. 34.48%) patients compared to the non-survivors (each *p* < 0.001). In addition, survivors had a lower incidence of cardiovascular disease (29.58% vs. 44.83%) and bowel ischemia (18.57% vs. 34.48%), both statistically significant with *p* values of 0.030 and 0.003, respectively. In addition, the need for dialysis was lower in survivors (60.00%) compared to non-survivors (75.86%, *p* < 0.001). In contrast to OS, there were significant differences in ICU-stay for the 100-day survival, with on average 44 days in the non-survivors and 98.07 days in the survivor group (*p* = 0.008). While overall hospital stays with 69.96 days in the non-survivors and 135.69 in the survivors did not differ significantly. Both first muscle loss and average muscle loss per day, with values of 1.84% vs. 3.54% and 0.73% vs. 1.61% in survivors vs. non-survivors, respectively, showed statistically significant differences (*p* = 0.022 and *p* < 0.001). These results are compiled in Table 6.

**Table 6:** Compiled are the significantly diverging variables between survivors and non-survivors for overall and 100-day survival

Group	Category		Mean %	Sig
Overall survival	Overweight	S	47.46	*p* = 0.017
		NS	65.85	
	Obese	S	11.86	*p* < 0.001
		NS	29.27	
	Cardio-vascular	S	28.81	*p* = 0.022
		NS	41.46	
	Bowl ischemia	S	17.24	*p* = 0.001
		NS	31.71	
	Dialysis	S	56.90	*p* < 0.001
		NS	75.61	
(S = 59; NS = 41)	First loss rate	S	− 1.86	*p* = 0.075
		NS	− 3.02	
	AVG Loss rate	S	− 0.74	*p* < 0.001
		NS	− 1.34	
100-day survival	Overweight	S	47.89	*p* < 0.001
		NS	72.41	
	Obese	S	12.68	*p* < 0.001
		NS	34.48	
	Cardio-vascular	S	29.58	*p* = 0.030
		NS	44.83	
	Bowl ischemia	S	18.57	*p* = 0.003
		NS	34.48	
	Dialysis	S	60.00	*p* < 0.001
		NS	75.86	
(S = 59; NS = 41)	First Loss rate	S	− 1.84	*p* = 0.022
		NS	− 3.54	
	AVG Loss rate	S	− 0.73	*p* < 0.001
		NS	− 1.61	

**Fig. 3:**
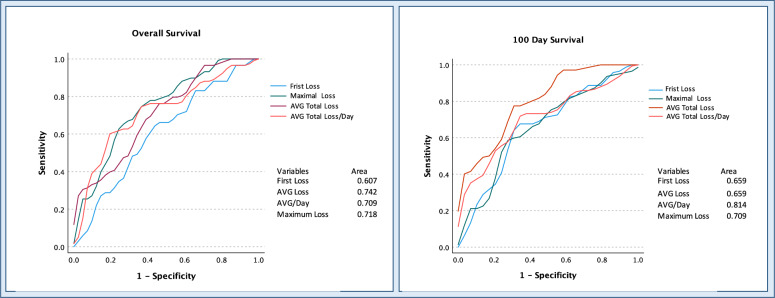
ROC analysis of multiple muscle decay rates for 100 days and overall survival. Naturally, the average daily muscle decay over the entire stay showed good discriminatory power for survival. However, the values of the first and maximum muscle loss, available much earlier during hospitalization, also showed good predictive power

ROC analysis was applied to assess the ability of different muscle loss rates to predict survival outcomes, overall and 100-day survival. Concerning OS, the first loss rate showed moderate predictability (AUC: 0.607, *p* = 0.054), the maximal decay rate showed slightly better performance (AUC: 0.718, *p* = 0.016). The relative total/long-term loss and relative total loss per day showed AUC values of 0.742 (*p* < 0.001) and 0.709 (*p* < 0.001) respectively. Focusing on muscle loss rates and 100-day survival outcomes, the first incurred decay rate showed moderate predictability (AUC: 0.659, *p* = 0.006), while the maximum loss rate per day showed better performance (AUC: 0.709, *p* < 0.001). Total/long-term loss showed a comparable predictability AUC: 0.659 (*p* = 0.013). Notably, long-term average loss per day stood out as having the highest predictive potential among the variables, with a substantial AUC of 0.814 (*p* < 0.001), indicating robust predictive ability for 100-day survival outcomes (Fig. 3).

**Fig. 4:**
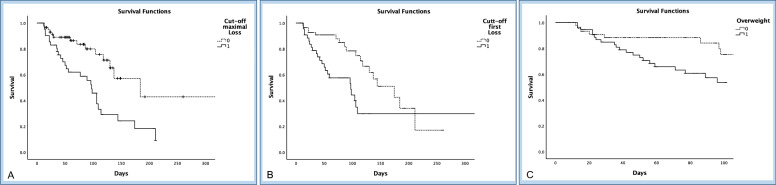
Kaplan–Meier curves of overall survival in ICU-patients with severe pancreatitis. Individuals were grouped according to a cut off for the maximal (**A**) and first (**B**) occurred muscle decay. Muscle loss beyond the defined thresholds showed significantly reduced survival (*p* = 0.001 and *p* = 0.013). In addition, survival was significantly limited in overweight (BMI > 25 kg/m.^2^) patients (C; *p* = 0.037)

Based on the ROC tables, we used the Youden index calculation to determine optimal thresholds for OS and found a maximum muscle loss rate of 3.23% per day, as well as a first loss rate of 1.98% per day. Utilizing these determined cutoff values and considering the prevalence of overweight individuals in the patient cohort, we conducted a Kaplan–Meier analysis. The results indicated notable disparities in OS among patients exceeding both first muscle loss (*p* = 0.013) and the maximum muscle loss cutoff (*p* = 0.001). Furthermore, individuals identified as overweight at ICU admission exhibited significantly reduced 100-day survival rates (*p* = 0.037). Results are shown in Fig. 4.

## Discussion

Securing positive outcomes in ICU patients with severe pancreatitis poses a distinct interdisciplinary challenge to health care providers due to the disease's unpredictable courses and the potentially fatal complications. In the present patient collective mortality was high at 41% and patients experienced prolonged hospitalization (mean: 116.63 days; ICU: 82.39 days) and numerous complications, including renal failure requiring dialysis (64 patients), bleedings (51), bowel ischemia (23) and abdominal compartment syndrome (12). The proportion of overweight individuals (55%) in our cohort was only slightly above the proportion in the general population [14], while the prevalence of sarcopenia (42%), was below reported averages of 60–70% in critically ill patients [15]. In the subgroup analysis, we were able to determine distinct differences between patient groups. Comparison of OS groups (59 survivors and 41 non-survivors) revealed notable deviations in pre-existing conditions and complications. The prevalence of overweight and obesity (p = 0.017 and *p* < 0.001, respectively), as well as cardiovascular disease, intestinal ischemia, and the requirement for dialysis, was higher among non-survivors (*p* = 0.022, *p* = 0.001, and *p* < 0.001, respectively). In addition, we categorized the cohort into five pancreatitis etiology-based groups: post-ERCP (*n* = 22), ethyl-toxic (*n* = 23), biliary (*n* = 23), post-surgical and other (*n* = 19), and unknown cause (*n* = 13). Interestingly, the biliary group, post-surgical and other, as well as those patients with an unknown cause of pancreatitis had a notably lower survival rates, compared to post-ERCP (68%, *p* = 0.008) and ethyl-toxic groups (83%, *p* < 0.001). The biliary group, with the lowest survival rate also comprised the lowest proportion of females and significantly older patients (*p* = 0.038, *p* = 0.002).

The main objective of this study was to characterize the long-term muscle loss in a largely uniform cohort of patients with ICU-requiring pancreatitis. This aimed at demonstrating the potential for a low-cost and low-effort muscle-monitoring approach and drawing potential implications for clinical care from the collected data. To this end, we applied an AI-tool for the segmentation of PMA in clinically indicated CT scans. This method revealed variable and patient-specific rates of muscle loss, with a non-linear progression characterized by an overall negative trend with different rates of decline at different timepoints. No scans had to be excluded due to quality limitations. The average muscle loss observed throughout the clinical course was 48.20% (CI 33.87–58.73%) of the baseline. The mean initial loss, recorded on average 6.16 days after ICU admission, amounted to 2.33% per day (CI 1.73–2.93%), which aligns with a recent meta-analysis involving 3251 ICU patients that reported a 2% daily muscle loss during the first week following admission [7]. Diverging from other studies, our research revealed that the peak rate of muscle loss does not always manifest within the initial days. In our cohort, this maximum rate occurred, on average 17.03 days after admission to the ICU. Importantly, it surpassed the initial loss rates, registering an average of 4.63% per day in survivors (CI 3.38–5.87%) and 5.54% (CI 4.01–7.07%) per day in non-survivors. In most cases, the loss rate stagnated over the longer period, resulting in a long-term rate averaging 0.98% (CI 0.82–1.14%) per day. The average muscle loss over the first 100 days and over the entire hospital stay was strongly associated with survival (AUC 0.709 and 0.814, respectively). Both decay rates, first and maximum loss per day, exhibited a robust discriminatory capacity for predicting survival (both 100 days and overall survival) in ROC analysis, with AUCs of 0.718 and 0.709 for the maximal decay rate, and AUCs of 0.607 and 0.659 for the first loss. Utilizing the ROC tables, we determined thresholds for overall survival for maximum muscle loss (3.23% per day) as well as the first loss (1.98% per day). Patients with muscle loss beyond these values had significantly limited survival probabilities (*p* = 0.001 and *p* = 0.013).

While the dynamics of muscle loss in the first few days and its association with survival have been extensively studied in prior research, there is limited knowledge on long-term muscle decay. Our research stands out by introducing distinctive and novel findings that advance this field [16, 17]. First, the measurements in our study are derived exclusively from clinically indicated CT scans, as opposed to the vast majority of study designs that adhere to predetermined measurement intervals over the first 2 weeks [7]. While this approach results in a lower resolution of day-to-day changes, it provides coverage throughout hospitalization. This enabled us to identify the peak rates of muscle decay, even in those cases where patients experienced it beyond the first weeks after admission to the ICU. Second, the majority of studies on muscle decay deployed ultrasound assessments. These benefit from their convenient bedside availability, but are contingent upon the presence of medical personnel and limited by issues related to reproducibility and errors [18, 19]. In contrast, our presented methodology integrates an AI-based segmentation tool, offering a convenient and low-effort applicability that could be easily integrated into clinical routines. Third, a prevalent issue in many prior studies on muscle wasting lies in the inclusion of markedly heterogeneous and small cohorts. Concentrating on a specific patient entity and lager study populations presents the advantage of enabling focused subgroup analyses. This approach aids in discerning significant distinctions not only among survival groups but also across diverse disease etiologies and patient-specific factors—such as sarcopenia or obesity in our case. The data presented here not only affirm the association of obesity as a risk factor in pancreatitis patients [20], but also intriguingly reveal that obese individuals experience a notably greater extent of muscle loss than their normal-weight or sarcopenic counterparts. In addition, the sub-group analysis highlighted particularly severe outcomes in patients with biliary pancreatitis, demonstrating a mortality rate of 70% and a maximum daily muscle loss of 7.79%. Our findings align with a comprehensive European study, affirming that ethyl toxic and biliary pancreatitis are the most prevalent causes. Despite this concurrence, the mentioned study did not uncover any survival differences among the etiological groups. However, it is crucial to emphasize that, unlike our study, this particular investigation did not exclusively concentrate on ICU patients [21].

Envisioning the routine integration of our method, which would impose only a minimal burden on clinically indicated CT scans, opens up multiple and versatile applications. Based on our data, additional information about the extent of muscle loss suffered and the associated prognosis could prove valuable in making informed decisions and thus contribute to personalized patient care. This might be particularly valuable when assessing the likelihood of survival is challenging, considering that conventional scores, such as the SOFA score, sometimes exhibit limited performance [22], whereas the extend of muscle decay reflects a robust estimate of survival probabilities. Besides the envisaged diagnostic values of CT imaging, the here presented method adds a layer of insight that substantiates the awareness on patient’s nutritional status. Prior research has highlighted the significant clinical advantages of early nutritional support in AP patients, including reduced mortality, organ failure, and infectious complications. However, assessing nutritional status remains challenging. While traditional anthropometric and biochemical markers may be difficult to obtain, changes in tissue mass is recommended as a more reliable indicator [5, 6]. Monitoring the tissue dynamics of ICU patients, even if intermittently, will help to identify those individuals suffering from extensive catabolism.

Another application area involves the early detection of patients at risk of post-intensive care syndrome (PICS). This umbrella term encompasses the occurrence of mid-to-long-term cognitive, psychological, and/or physical impairments in patients after ICU stays [23, 24]. Intensive care acquired weakness (ICUAW), a component of PICS, leads to significant muscle wasting, affecting peripheral and respiratory muscles. Recent studies underscore the considerable impact of ICUAW on post-discharge quality of life, emphasizing the crucial need for early identification of at-risk patients [25–27]. Despite the absence of a proven treatment [28], our method could serve as a valuable tool for identifying severe muscle decay, guiding the assessment of muscle strength, and facilitating preventive support for these patients.

### Limitations

Given the retrospective nature of our study, a selection bias is inevitable. Due to the inclusion criteria, less severe patients are presumably underrepresented. In addition, the retrospective design of the study poses challenges in establishing causality between the degree and timing of muscle wasting and the onset of complications or clinical deterioration, and in assessing the potential impact of factors, such as neuromuscular blockers or parenteral nutrition. While s*ingle-slice assessment at the L3 level is currently considered the gold standard in clinical practice* [11]*, muscle volume assessment may provide additional insights. It is important to note that muscle area assessment alone does not directly reflect muscle function, and thus, conclusions regarding functionality cannot be extrapolated.* Future prospective studies with a more robust design could provide a clearer understanding of the dynamics of muscle loss rates.

## Conclusion

The innovative AI-based PMA segmentation method proved to be a robust and effortless monitoring tool, allowing us to be the first to present a comprehensive assessment of muscle wasting in a big cohort of intensive care patients with pancreatitis. Our findings reveal significant muscle wasting among these patients, averaging 48.20%, with a pronounced impact observed in overweight individuals. Notably, higher rates of initial and maximum muscle loss, detectable early in the clinical trajectory, exhibited a strong correlation with survival. Integration of this AI-based monitoring tool into routine clinical practice will enable ongoing tracking of patients' muscle status and, in some cases, facilitate early identification of individuals at risk of poor outcomes or in need for intensified support.

## Data Availability

The data sets generated during and analyzed during the current study are available from the corresponding author on reasonable request.

## Abbreviations

AI: Artificial intelligence
ANOVA: Analysis of variance
AP: Acute pancreatitis
BIA: Bioelectrical impendence analysis
BMI: Body mass index
CI: Confidence interval
CT: Computed tomography
ECMO: Extracorporeal membrane oxygenation
ICU: Intensive care unit
OS: Overall survival
PACS: Picture archiving and communication system
PICS: Post-intensive care syndrome
PMA: Psoas muscle area
SAT: Subcutaneous adipose tissue
SMA: Skeletal muscle area
SOFA: Sequential organ failure assessment
TAMA: Total abdominal muscle area
US: Ultrasound
VAT: Visceral adipose tissue
